# Prevalence and severity of pediatric emergencies in a German helicopter emergency service: implications for training and service configuration

**DOI:** 10.1007/s00431-023-05178-8

**Published:** 2023-09-01

**Authors:** Stefan Mockler, Camilla Metelmann, Bibiana Metelmann, Karl Christian Thies

**Affiliations:** 1 Department of Anesthesiology, University Hospital Greifswald, Sauerbruchstr, 17475 Greifswald, Germany; 2 Department of Anaesthesiology and Critical Care, EvKB, OWL University Medical Center, Campus Bielefeld Bethel, Burgsteig 13, 33617 Bielefeld, Germany

**Keywords:** Pediatric emergency medicine, Epidemiology of pediatric emergencies, Emergency medical service, Pediatric emergency treatment, Pediatric airway management

## Abstract

This study primarily aims to determine the frequency of life-threatening conditions among pediatric patients served by the DRF, a German helicopter emergency service (HEMS) provider. It also seeks to explore the necessity of invasive procedures in this population, discussing the implications for HEMS crew training and service configuration based on current literature. We analyzed the mission registry from 31 DRF helicopter bases in Germany, focusing on 7954 children aged 10 or younger over a 5-year period (2014–2018). Out of 7954 identified children (6.2% of all primary missions), 2081 (26.2%) had critical conditions. Endotracheal intubation was needed in 6.5% of cases, while alternative airway management methods were rare (n = 14). Half of the children required intravenous access, and 3.6% needed intraosseous access. Thoracostomy thoracentesis and sonography were only performed in isolated cases.

*  Conclusions*: Critically ill or injured children are infrequent in German HEMS operations. Our findings suggest that the likelihood of HEMS teams encountering such cases is remarkably low. Besides endotracheal intubation, life-saving invasive procedures are seldom necessary. Consequently, we conclude that on-the-job training and mission experience alone are insufficient for acquiring and maintaining the competencies needed to care for critically ill or injured children.

**Table:** 

**What is Known:** *• Pediatric emergencies are relatively rare in the prehospital setting, but their incidence is higher in helicopter emergency medical services (HEMS) compared to ground-based emergency services.*	
**What is New:** *• On average, HEMS doctors in Germany encounter a critically ill or injured child approximately every 1.5 years in their practice, establish an IV or IO access in infants or toddlers every 2 years, and intubate an infant every 46 years.* *• This low frequency highlights the insufficiency of on-the-job training alone to develop and maintain pediatric skills among HEMS crews. Specific interdisciplinary training for HEMS crews is needed to ensure effective care for critically unwell pediatric patients.*

**What is Known:**

*• Pediatric emergencies are relatively rare in the prehospital setting, but their incidence is higher in helicopter emergency medical services (HEMS) compared to ground-based emergency services.*

**What is New:**

*• On average, HEMS doctors in Germany encounter a critically ill or injured child approximately every 1.5 years in their practice, establish an IV or IO access in infants or toddlers every 2 years, and intubate an infant every 46 years.*

*• This low frequency highlights the insufficiency of on-the-job training alone to develop and maintain pediatric skills among HEMS crews. Specific interdisciplinary training for HEMS crews is needed to ensure effective care for critically unwell pediatric patients.*

## Introduction

Physician-staffed helicopter emergency medical services (HEMS) are expected to provide high-quality care to patients of all age groups, including children. International data indicates that pediatric patients make up approximately 5–10% of HEMS missions, although this figure varies depending on the age range defined as “pediatric” and the specific patient population served [1–4]. In the USA, medical procedures were performed on 50% of children encountered by regular emergency medical services (EMS), but critical procedures were only carried out in 1.5% of cases [1, 5]. HEMS, however, have reported higher rates of critical medical interventions, such as endotracheal intubation (ETI), ranging from 3.9 to 18.8% [2, 3, 6–9]. Some studies suggest that HEMS transportation for seriously injured children may be associated with improved survival outcomes [10].

Nonetheless, there is limited information on the severity and types of emergencies encountered by German HEMS, as well as the frequency of invasive procedures performed in prehospital settings. The primary objective of this study is to determine the prevalence of life-threatening conditions among patients served by a prominent German HEMS provider.

The secondary objective of the study is to ascertain the frequency of invasive procedures performed on this patient population. Additionally, we will examine the implications for HEMS crew training and service configuration, considering the existing literature on the subject.

## Materials and methods

We undertook a retrospective registry study to analyze de-identified patient data using quantitative methods.

### Study setting

In Germany, EMS encompass ground emergency medical services (GEMS), which include ambulances and doctor-staffed intervention vehicles, as well HEMS. The HEMS team consists of a pilot, an emergency medical technician, and a doctor. Based on the incident’s severity and location, regional EMS dispatchers determine the most appropriate response. This study involves a retrospective analysis of mission registry data from a major German HEMS provider, DRF Luftrettung, which operates 31 of Germany’s 90 helicopter posts.

### Data acquisition

The electronic registry comprises anonymized patient records for all DRF Luftrettung-operated helicopter posts. After each mission, HEMS crews enter the data at their respective posts. All staff members receive training in database operation and accurate data entry. Mandatory data fields of interest minimize missing information. Our study includes all medical data for patients younger than 11 years old, attended to between January 1, 2014, and December 31, 2018. We chose the 11th birthday as the cut-off for inclusion, because beyond this age, anatomy and physiology more closely resemble adult conditions [11]. We excluded datasets from secondary missions (i.e., transfers between two hospitals). Data was extracted in an aggregated format rather than on individual case basis.

### Statistical analysis

We grouped pediatric patients into four categories for comparison: infants (less than 1 year old), toddlers (1 to 3 years old), preschoolers (4 to 5 years old), and primary school pupils (6 to 10 years old). We employed parametric tests, such as *t*-tests and *χ*^2^-tests, to compare groups. The significance level was set at *p* < 0.05.

## Results

### Included cases

Between 2014 and 2018, DRF attended to 127,964 primary missions. A total of 7954 (6.2%) patients were aged 10 years or younger. Among these pediatric cases, 14.3% (*n* = 1137) were infants (less than 1 year old), 39.7% (*n* = 3157) were toddlers (1 to 3 years old), 13.2% (*n* = 1053) were preschoolers (4 to 5 years old), and 32.8% (*n* = 2607) were primary school children (6 to 10 years old). The gender distribution of the included cases is presented in Table 1.

**Table 1:** Gender distribution per age group

***Gender***	*** < 1 year*** ***n = 1137 (%)***	***1–3 years old n = 3157 (%)***	***4–5 years old n = 1053 (%)***	***6–10 years old n = 2607 (%)***
***Female***	*513 (47.46)*	*1299 (42.96)*	*407 (40.42)*	*991 (39.67)*
***Male***	*568 (52.54)*	*1725 (57.04)*	*600 (59.58)*	*1507 (60.33)*
***Not known***	*56*	*133*	*46*	*109*

### Severity of illnesses and injuries

**Fig. 1:**
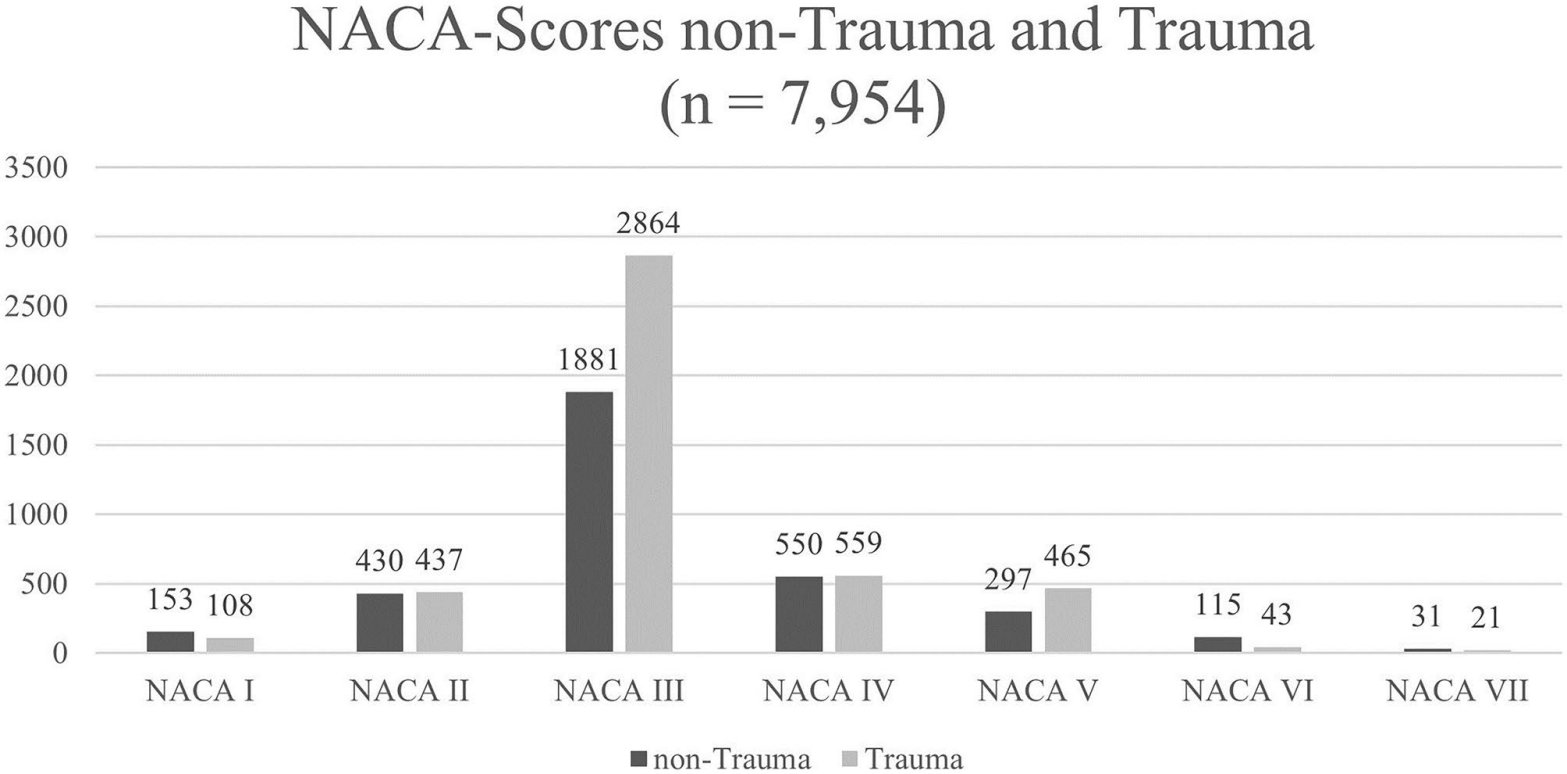
NACA score for non-trauma and trauma patients

In Germany, EMS evaluates the severity of illnesses and injuries using the National Advisory Committee for Aeronautics (NACA) score [12]. Table 2 shows the seven NACA score levels with the respective severity. Table 3 displays the NACA score levels across the four age groups. The proportion of life-threatening conditions (NACA IV-VII) was highest among infants (33.9%), followed by preschoolers (27.5%), toddlers (25.0%), and primary school children (23.7%). Figure 1 illustrates the distribution of injured and ill children with respect to NACA scores and age.

**Table 2:** NACA score

**NACA score**	
NACA I	Minor condition
NACA II	Medical treatment required
NACA III	Hospital admission required
NACA IV	Life threatening condition cannot be excluded
NACA V	Life-threatening condition
NACA VI	Cardiac arrest with return of spontaneous circulation or transport to hospital under resuscitation
NACA VII	Lethal condition

**Table 3:** NACA score levels per age group

**NACA score**	** < 1 year** ***n* = 1137 (%)**	**1–3 years old** ***n* = 3157 (%)**	**4–5 years old** ***n* = 1053 (%)**	**6–10 years old ** ***n* = 2607 (%)**
NACA I	56 (4.93)	95 (3.01)	24 (2.28)	86 (3.3)
NACA II	125 (10.99)	317 (10.04)	115 (10.92)	310 (11.89)
NACA III	571 (50.22)	1955 (61.93)	625 (59.35)	1594 (61.14)
NACA IV	197 (17.33)	449 (14.22)	161 (15.29)	302 (11.58)
NACA V	123 (10.82)	264 (8.36)	106 (10.07)	269 (10.32)
NACA VI	46 (4.05)	63 (2.00)	17 (1.61)	32 (1.23)
NACA VII	19 (1.68)	14 (0.44)	5 (0.47)	14 (0.54)

### Prevalence of cardiac arrest

In total, 210 children experienced cardiac arrest (NACA scores VI or VII). Among these cases, 64 were due to trauma, 30 resulted from drowning, and 5 occurred peri-partum. Sudden infant death syndrome was suspected in 10 instances. For 52 patients (24.8%) who suffered cardiac arrest, they were declared deceased either at scene or during transport.

### Prevalence of altered vital signs

In 86.5% (*n* = 6881), the oxygen saturation levels were recorded. At consultation, 2.0% (*n* = 137) had 85–89% saturation, and 1.9% (*n* = 131) had < 85%. At hospital handover, these numbers significantly decreased to 0.3% (*n* = 20) and 0.4% (*n* = 27) (*p* < 0.001).

Glasgow Coma Scale (GCS) was documented in all cases. GCS scores of 9–12 were observed in 7.3% (*n* = 580) of children initially and 6.2% (*n* = 492) at handover. GCS scores of ≤ 8 was found in 9.2% (*n* = 729) initially and 8.9% (*n* = 709) at handover. GCS improvements were significant (*p* = 0.022).

In 45% (*n* = 3578) of children, heart rhythm was documented. Pathological heart rhythms were observed in 6.3% (*n* = 227) of cases, including asystole (*n* = 87), narrow QRS complex tachycardia (*n* = 61), pulseless electrical activity (*n* = 26), atrial arrhythmia, (*n* = 15), wide QRS complex tachycardia (*n* = 12), bundle branch block (*n* = 7), pacemaker (*n* = 7), acute ischemic changes (*n* = 4), ventricular fibrillation (*n* = 3), atrioventricular block (*n* = 3), and extrasystoles (*n* = 2).

### Non-trauma conditions

Out of the 7954 patients, 3457 (43.5%) presented with a non-trauma condition. A diagnosis was documented in 3373 cases (97.6%). Table 4 displays the prevalence of prehospital non-trauma diagnostic categories alongside their corresponding NACA scores. In 1494 instances (44.2%), children presented with seizures or febrile convulsions as their primary diagnoses.

**Table 4:** Prehospital non-trauma diagnostic categories and NACA score levels in order of frequency of appearance (*n* = 3373)

**Category of diagnosis**	**Total number (%)**	**NACA I-III n, (%)**	**NACA IV-VII n, (%)**
**Neurological, neurosurgical**	1624 (48.15)	1189 (73.21)	435 (26.79)
**Breathing, airway**	684 (20.28)	449 (65.64)	235 (34.36)
**Gastrointestinal tract**	185 (5.48)	168 (90.81)	17 (9.19)
**Anaphylaxis**	175 (5.19)	146 (83.43)	29 (16.57)
**Cardiac condition**	148 (4.38)	31 (20.94)	117 (79.04)
**Circulation (excluding infection)**	137 (4.06)	121 (88.32)	16 (11.68)
**Intoxication**	114 (3.38)	92 (80.70)	22 (19.30)
**Infection**	90 (2.67)	69 (76.67)	21 (23.33)
**Near-drowning**	64 (1.90)	15 (23.44)	49 (76.56)
**Psychiatric**	59 (1.75)	59 (100.00)	0 (0.00)
**Metabolic/endocrinologic**	30 (0.89)	14 (46.67)	16 (53.33)
**Transition after birth**	23 (0.68)	9 (39.13)	14 (60.87)
**Other**	40 (1.19)	30 (75.00)	10 (25.00)

### Trauma

**Fig. 2:**
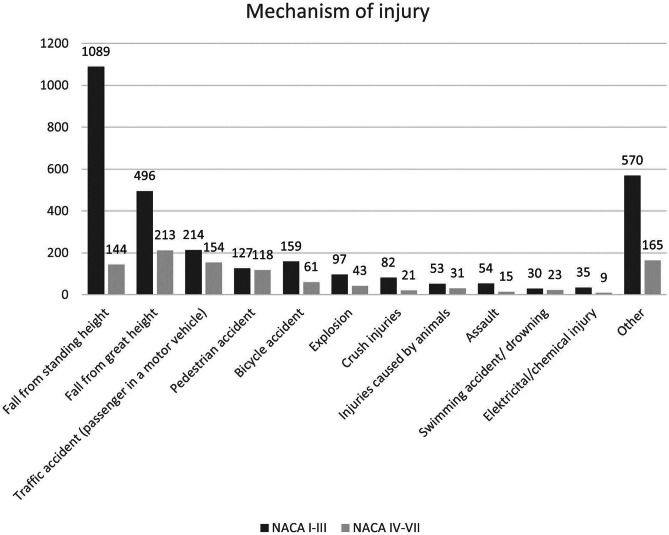
Mechanism of injury

In 4497 cases (56.5%), HEMS responded to incidents involving injuries and trauma. The primary trauma mechanisms were falls (*n* = 1942), followed by road traffic accidents (*n* = 833). Polytrauma was documented in 219 cases (4.9%). In 3507 cases, the affected body regions were documented, as illustrated in Table 5. Figure 2 displays the mechanism of injury.

**Table 5:** Injured body region by frequency. In 3507 patients, 4733 injuries were documented. Multiple selections per patient were possible

**Body region**	***n*= 4733**	**%**
**Head**	1651	34.9
**Limbs**	1.418	30.0
**Spine**	464	9.8
**Face**	374	7.9
**Chest**	307	6.5
**Abdomen**	288	6.1
**Pelvis**	149	3.1
**Neck**	82	1.7

Scalds and burns were in 662 the cause for alerting the HEMS. In a critical condition were 168 burned children (25.4%) (NACA IV-VII). Burned children (80.1%) were younger than 4 years.

### Prehospital interventions

Table 6 displays the prehospital interventions performed on children within the different age groups.

**Table 6:** Prehospital interventions per age group

**Intervention**	**< 1 year* n* = 1137 (%)**	**1–3 years old* n*= 3157 (%)**	**4–5 years old* n* = 1053 (%)**	**6–10 years old* n* = 2607 (%)**
Endotracheal intubation	68 (6.0)	194 (6.2)	77 (7.3)	181 (6.9)
IV access	327 (28.76)	1184 (37.50)	614 (58.31)	1887 (72.38)
IO access	85 (7.48)	148 (4.69)	20 (1.90)	30 (1.15)
Cervical spine immobilization	60 (5.3)	293 (9.3)	258 (24.5)	974 (37.4)
Thoracic and lumbar spine immobilization	63 (5.5)	186 (5.9)	207 (19.7)	879 (33.7)
Pelvic binder	2 (0.2)	9 (0.3)	8 (0.8)	23 (0.9)
(Compression) bandage	127 (11.2)	513 (16.2)	223 (21.2)	556 (21.3)
Tourniquets	1 (0.1)	1 (0.03)	0	11 (0.4)
Fracture realignment + joint reduction	9 (0.8)	36 (1.1)	46 (4.4)	213 (8.2)
Splinting	27 (2.4)	112 (3.5)	143 (13.6)	539 (20.7)

ETI was performed on 520 patients (6.5%). Video laryngoscopy (which was not part of the standard equipment during the study period) was used in 16 cases to secure the airway, while laryngeal masks were employed in 2 cases and laryngeal tubes in 12. Surgical airway procedures were carried out in 2 cases. Needle thoracocentesis was performed in 3 cases, and chest drains were established in 8 cases.

In total, 22 central lines and 6 arterial lines were established. Prehospital sonography was performed in 155 cases (1.9%).

Regrettably, the administration of medication could not be traced back to individual cases. The numbers provided represent the total sum of all drug administrations. For patients presenting with non-trauma conditions, there were 2552 drug administrations in total, with 1297 (50.8%) given to patients with NACA IV or higher. For patients with trauma, there were 5408 drug administrations in total, with 2304 (42.6%) given to patients with NACA IV or higher. The most frequently given drugs were sedatives, non-opioid analgesics, opioid analgesics, anesthetics, muscle relaxants, antiemetics, and inotropes.

### Transport to hospital

Among all pediatric patients, 7403 (93.1%) were transported to the hospital. Of these, 3998 (54.0%) were transported via helicopter, 1976 (26.7%) by ambulance accompanied by a helicopter physician, and 1429 (19.3%) by ambulance without a helicopter physician.

## Discussion

Children constitute a small proportion (6.2%) of patients encountered by German HEMS. Only 26.1% of these children are critically unwell (NACA IV-VII), and 6.5% require an ETI. For an average German HEMS post with 20 doctors flying 830 primary missions annually, this equates to one seriously unwell child aged 10 or younger per doctor per 18 months and one ETI every 6 years. For children 1 year or younger, this translates to one ETI in 46 years. Safe airway management is a crucial skill in pediatric resuscitation. A recent meta-analysis found intubation failure to be over three times more likely in pediatric patients compared to adults [13]. Prehospital tube misplacement rates range from 0.3 to 26.2% in various studies [13–18]. A significant risk factor appears to be low body weight. In a pediatric patient population, Rost et al. found a lower tube misplacement rate (composite outcome: unrecognized esophageal, endobronchial, or too deep ETI) on hospital admission when ETI was performed by HEMS (23.5%) compared to GEMS (64.7%) crews [18]. To successfully manage pediatric airways, especially in the youngest age group, specific training is necessary. Well-trained staff (> 2500 ETI) exhibit low complication rates for pediatric tracheal intubations in a prehospital setting [17, 19]. In a retrospective analysis of intubation competence among neonatal-perinatal medicine fellows, it was shown that the number of neonatal intubations required to achieve procedural competence varied, with a minimum of 8–46 supervised neonatal endotracheal intubations needed to reach an overall success rate of 80% within two attempts [20]. Foglia demonstrated in an international registry study that neonatal intubation success in neonatal intensive care units varied among provider levels, with a first-attempt success rate of 24% for pediatric residents and 64% for attending neonatologists [21]. The first-attempt success rate is increased by using a video laryngoscope [22, 23]. It can be assumed that even with training, emergency physicians with a non-pediatric background will not exceed the level of pediatric residents. Opportunities to achieve pediatric ETI competencies are becoming increasingly limited [24], likely due to the shift in pediatric anesthesia practice from ETI to supraglottic airways. However, there are also effective alternatives to ETI for prehospital airway management, such as bag valve mask ventilation or supraglottic airways [25].

The youngest age group contained the highest proportion of critically unwell children (33.9%), yet it is surprising that only 29% of patients in this group had vascular access established. In contrast, in the other age groups, the percentage of IV access increased successively to 72% despite falling NACA scores (Table 4). This observation does not clearly indicate either undertreatment in infants or overtreatment in older age groups. It is possible that the discrepancy could be attributed to a higher incidence of respiratory diseases in infants, which may not require vascular access but rather call for inhalation and oral/rectal steroids. The IO access rate is highest at 7% in the youngest age group and falls to 1% in the oldest age group. In general, IO access is an easy method to become proficient in [26], with a first-attempt success rate of about 80% [27]. However, a high failure rate (53%) has been reported in a post-mortem CT analysis in children younger than 6 months [28].

Rogers et al. found that over one-third of EMS practitioners expressed a low comfort level in managing critically ill children [29]. Increasing continuing education in pediatric emergencies enhances the level of comfort felt by prehospital providers when treating pediatric patients [29–31].

Aside from increasing difficulties in achieving advanced pediatric competencies, it is also unclear how acquired competencies can be maintained. Previous studies have shown a significant loss of knowledge and a reduction in training effect just 3 months after attending a single neonatal resuscitation course [32]. When learning units are repeated over a more extended period, the acquired knowledge and skills seem to persist longer [32–34]. A meta-analysis by Legoux et al. demonstrated a decrease in skills learned in simulation after 3, 6, and 12 months, but still above the baseline before course attendance [35]. Yang et al. showed a decrease in skills learned for certified life support courses before the two-year recertification deadline [36, 37]. In a long-term observational study, Ansquer had French emergency physicians undergo pediatric emergency simulation training and observed a drop in competence after 6 months and a total loss of the trained competencies after 4 years [38].

Rarely required life-saving procedures, such as invasive airway maneuvers or chest drain insertion, are only rudimentarily taught in certified emergency courses. Additionally, it is unclear whether skills learned in a life support course are transferable to real-life practice [33]. Whether advanced simulation within a curricular framework can fully replace clinical training is also debatable [39]. Surveys of American pediatricians reveal that it is extremely challenging even for pediatricians to acquire and maintain these competencies [40].

The current legal pediatric training requirements for prehospital doctors in Germany range from 2 to 4 h, which we feel is insufficient to address the needs of seriously ill or injured children adequately. This disparity creates a gap between the necessary expertise for treating pediatric patients and the actual training provided. Considering the extensive training required to develop and maintain pediatric skills, it is questionable whether Helicopter Emergency Medical Service teams can attain appropriate expertise levels without regular involvement in pediatric anesthesia or critical care.

To bridge this gap, some healthcare systems, such as the British National Health Services, have established pediatric retrieval networks [19, 41]. Within these networks, high-volume pediatric centers provide telephone support and dispatch dedicated critical care teams to assist ambulance services and district general hospitals with the initial care for critically ill children. In other systems, such as Emergency Medical Services Munich, Germany, hospitals send rapid response vehicles with pediatric critical care physicians to support ambulance services when responding to cases involving critically ill children [42, 43]. Both service configurations enable the delivery of highly professional care on scene and during transport. However, the availability of such systems in Germany is limited. For the foreseeable future, prehospital care for children will primarily be provided in an integrated model, delivered by practitioners who only treat children occasionally. Therefore, specific pediatric training programs, going beyond the level of life support courses, are necessary to enable these practitioners to assess unwell children properly and perform critical interventions safely. Such programs could consist of blended learning, advanced simulation training, and clinical sessions, where HEMS teams perform critical interventions in a hospital setting under supervision. Given the predictable decline of knowledge and skills over time, regular refresher trainings should be mandatory to ensure the teams sustain their pediatric expertise.

Furthermore, HEMS should seek collaborations with regional pediatric centers. These partnerships can strengthen clinical governance, provide hospital training opportunities, and offer telemedical support to HEMS crews on the scene or during transport, if needed.

## Limitations

This retrospective analysis focuses on data collected from a single Helicopter Emergency Medical Services operator in Germany, which may affect the generalizability of the results. Despite this limitation, the dataset accounts for approximately 35% of all HEMS missions conducted in the country during the study period. Considering the broad geographical distribution of the posts involved, it is reasonable to assume that the data provides a representative snapshot of the pediatric population encountered by HEMS on a national scale.

However, it is important to note that due to the German interpretation of the European Data Protection Act, the researchers were unable to correlate the data with hospital outcomes. This restriction prevents the study from providing a comprehensive understanding of the impact of HEMS interventions on patient outcomes, limiting the ability to draw strong conclusions about the overall effectiveness of HEMS in pediatric care and the quality of care delivered.

While the entry screen of the database required all essential fields such as age, GCS, NACA, and diagnosis to be completed, we cannot confirm if all other fields were filled in accurately. Although no data is missing, there is a possibility of under-reporting due to incomplete or erroneous entries in other fields. HEMS teams were not obliged to report success of invasive procedures or number of attempts needed.

Physiological values were not obligatory and presented aggregated in groups. It is worth noting that these values were not customized to cater for children.

Further research involving multiple HEMS operators, as well as overcoming the limitations imposed by the European Data Protection Act, would be necessary to obtain a more comprehensive and generalizable view of HEMS in pediatric care across Germany. Nevertheless, the current study still offers valuable insights into the pediatric population served by HEMS and can serve as a starting point for future research and policy discussions.

## Conclusion

Critically ill children are a rarity in German HEMS, which will result in limited exposure for individual teams to genuine pediatric emergencies. Current training standards for prehospital care providers predominantly concentrate on the adult patient population, with minimal focus on children. German HEMS operator should feel encouraged, to invest in the creation of specialized pediatric training programs for advanced prehospital care teams. This will bridge the gap between the current training landscape and the aspiration to deliver exceptional care to all patients.

## Data Availability

Original data would be shared upon reasonable request.

## Abbreviations

EMS: Emergency medical service
ETI: Endotracheal intubation
GCS: Glasgow Coma Scale
GEMS: Ground emergency medical service
HEMS: Helicopter emergency medical service
IO: Intraosseus
IV: Intravenous
NACA: National Advisory Committee for Aeronautics

## References

[1] Drayna P, Browne L, Guse C, Brousseau D, Lerner E (2015). Prehospital pediatric care: opportunities for training, treatment, and research. Prehospital emergency care: official journal of the National Association of EMS Physicians and the National Association of State EMS Directors.

[2] Enomoto Y, Tsuchiya A, Tsutsumi Y, Kikuchi H, Ishigami K, Osone J, Togo M, Yasuda S, Inoue Y (2021). Characteristics of children cared for by a physician-staffed helicopter emergency medical service. Pediatr Emerg Care.

[3] Oude Alink M, Moors X, Karrar S, Houmes R, Hartog D, Stolker R (2021). Characteristics, management and outcome of prehospital pediatric emergencies by a Dutch HEMS. European journal of trauma and emergency surgery : official publication of the European Trauma Society.

[4] Rugg C, Woyke S, Ausserer J, Voelckel W, Paal P, Ströhle M (2021). Analgesia in pediatric trauma patients in physician-staffed Austrian helicopter rescue: a 12-year registry analysis. Scandinavian journal of trauma, resuscitation and emergency medicine.

[5] Carlson J, Gannon E, Mann N, Jacobson K, Dai M, Colleran C, Wang H (2015). Pediatric out-of-hospital critical procedures in the United States. Pediatric critical care medicine : a journal of the Society of Critical Care Medicine and the World Federation of Pediatric Intensive and Critical Care Societies.

[6] Eich C, Russo S, Heuer J, Timmermann A, Gentkow U, Quintel M, Roessler M (2009). Characteristics of out-of-hospital paediatric emergencies attended by ambulance- and helicopter-based emergency physicians. Resuscitation.

[7] Gerritse B, Schalkwijk A, Pelzer B, Scheffer G, Draaisma J (2010). Advanced medical life support procedures in vitally compromised children by a helicopter emergency medical service. BMC Emerg Med.

[8] Nielsen V, Bruun N, Søvsø M, Kløjgård T, Lossius H, Bender L, Mikkelsen S, Tarpgaard M, Petersen J, Christensen E (2022). Pediatric emergencies in helicopter emergency medical services: a national population-based cohort study from Denmark. Ann Emerg Med.

[9] Selig H, Trimmel H, Voelckel W, Hüpfl M, Trittenwein G, Nagele P (2011). Prehospital pediatric emergencies in Austrian helicopter emergency medical service — a nationwide, population-based cohort study. Wien Klin Wochenschr.

[10] Bläsius F, Horst K, Brokmann J, Lefering R, Andruszkow H, Hildebrand F, TraumaRegister D (2021) Helicopter emergency medical service and hospital treatment levels affect survival in pediatric trauma patients. J Clin Med 10(4). 10.3390/jcm10040837 10.3390/jcm10040837 PMC7922049 33670679

[11] Fleming S, Thompson M, Stevens R, Heneghan C, Plüddemann A, Maconochie I, Tarassenko L, Mant D (2011). Normal ranges of heart rate and respiratory rate in children from birth to 18 years of age: a systematic review of observational studies. Lancet (London, England).

[12] Schneider F, Martin J, Schneider G, Schulz C (2018) The impact of the patient’s initial NACA score on subjective and physiological indicators of workload during pre-hospital emergency care. PloS one 13(8):e0202215. 10.1371/journal.pone.0202215 10.1371/journal.pone.0202215 PMC6084954 30092090

[13] Rodríguez J, Higuita-Gutiérrez L, Carrillo Garcia E, Castaño Betancur E, Luna Londoño M, Restrepo Vargas S (2020). Meta-analysis of failure of prehospital endotracheal intubation in pediatric patients. Emergency medicine international.

[14] Burns B, Watterson J, Ware S, Regan L, Reid C (2017). Analysis of out-of-hospital pediatric intubation by an Australian helicopter emergency medical service. Ann Emerg Med.

[15] Eich C, Roessler M, Nemeth M, Russo S, Heuer J, Timmermann A (2009). Characteristics and outcome of prehospital paediatric tracheal intubation attended by anaesthesia-trained emergency physicians. Resuscitation.

[16] Matettore A, Ramnarayan P, Jones A, Randle E, Lutman D, O’Connor M, Chigaru L (2019). Adverse tracheal intubation-associated events in pediatric patients at nonspecialist centers: a multicenter prospective observational study. Pediatric critical care medicine : a journal of the Society of Critical Care Medicine and the World Federation of Pediatric Intensive and Critical Care Societies.

[17] Nevin D, Green S, Weaver A, Lockey D (2014). An observational study of paediatric pre-hospital intubation and anaesthesia in 1933 children attended by a physician-led, pre-hospital trauma service. Resuscitation.

[18] Rost F, Donaubauer B, Kirsten H, Schwarz T, Zimmermann P, Siekmeyer M, Gräfe D, Ebel S, Kleber C, Lacher M, Struck M (2022) Tracheal tube misplacement after emergency intubation in pediatric trauma patients: a retrospective, exploratory study. Children (Basel, Switzerland) 9(2). 10.3390/children9020289 10.3390/children9020289 PMC8870798 35205009

[19] Renberg M, Hertzberg D, Kornhall D, Günther M, Gellerfors M (2021). Pediatric prehospital advanced airway management by anesthesiologist and nurse anesthetist staffed critical care teams. Prehosp Disaster Med.

[20] Evans P, Shults J, Weinberg D, Napolitano N, Ades A, Johnston L, Levit O, Brei B, Krick J, Sawyer T, Glass K, Wile M, Hollenberg J, Rumpel J, Moussa A, Verreault A, Mehrem AA, Howlett A, Mc Kanna J, Nishisaki A, Foglia E (2021) Intubation competence during neonatal fellowship training. Pediatrics 148(1). 10.1542/peds.2020-036145 10.1542/peds.2020-036145 PMC8290971 34172556

[21] Foglia E, Ades A, Sawyer T, Glass K, Singh N, Jung P, Quek B, Johnston L, Barry J, Zenge J, Moussa A, Kim J, DeMeo S, Napolitano N, Nadkarni V, Nishisaki A (2019) Neonatal intubation practice and outcomes: an international registry study. Pediatrics 143(1). 10.1542/peds.2018-0902 10.1542/peds.2018-0902 PMC6317557 30538147

[22] Garcia-Marcinkiewicz AG, Kovatsis PG, Hunyady AI, Olomu PN, Zhang B, Sathyamoorthy M, Gonzalez A, Kanmanthreddy S, Gálvez JA, Franz AM, Peyton J, Park R, Kiss EE, Sommerfield D, Griffis H, Nishisaki A, von Ungern-Sternberg BS, Nadkarni VM, McGowan FX, Fiadjoe JE (2020). First-attempt success rate of video laryngoscopy in small infants (VISI): a multicentre, randomised controlled trial. Lancet (London, England).

[23] Lingappan K, Arnold JL, Fernandes CJ, Pammi M (2018) Videolaryngoscopy versus direct laryngoscopy for tracheal intubation in neonates. Cochrane Database Syst Rev 6(6):CD009975. 10.1002/14651858.CD009975.pub3 10.1002/14651858.CD009975.pub3 PMC6513507 29862490

[24] Marrs L, Zenge J, Barry J, Wright C (2019). Achieving procedural competency during neonatal fellowship training: can trainees teach us how to teach?. Neonatology.

[25] Tweed J, George T, Greenwell C, Vinson L (2018). Prehospital airway management examined at two pediatric emergency centers. Prehosp Disaster Med.

[26] Itoh T, Lee-Jayaram J, Fang R, Hong T, Berg B (2019). Just-in-time training for intraosseous needle placement and defibrillator use in a pediatric emergency department. Pediatr Emerg Care.

[27] Feldman O, Nasrallah N, Bitterman Y, Shavit R, Marom D, Rapaport Z, Kabesa S, Benacon M, Shavit I (2021). Pediatric intraosseous access performed by emergency department nurses using semiautomatic devices: a randomized crossover simulation study. Pediatr Emerg Care.

[28] Harcke H, Curtin R, Harty M, Gould S, Vershvovsky J, Collins G, Murphy S (2020). Tibial intraosseous insertion in pediatric emergency care: a review based upon postmortem computed tomography. Prehospital emergency care : official journal of the National Association of EMS Physicians and the National Association of State EMS Directors.

[29] Rogers C, Gausche-Hill M, Brown L, Burke R (2021). Prehospital emergency provider’s knowledge of and comfort with pediatric and special needs cases: a cross-sectional study in Los Angeles county. Eval Health Prof.

[30] Padrez K, Brown J, Zanoff A, Chen C, Glomb N (2021). Development of a simulation-based curriculum for pediatric prehospital skills: a mixed-methods needs assessment. BMC Emerg Med.

[31] Stevens S, Alexander J (2005). The impact of training and experience on EMS providers’ feelings toward pediatric emergencies in a rural state. Pediatr Emerg Care.

[32] Huang J, Tang Y, Tang J, Shi J, Wang H, Xiong T, Xia B, Zhang L, Qu Y, Mu D (2019). Educational efficacy of high-fidelity simulation in neonatal resuscitation training: a systematic review and meta-analysis. BMC Med Educ.

[33] Finan E, Bismilla Z, Campbell C, Leblanc V, Jefferies A, Whyte HE (2012). Improved procedural performance following a simulation training session may not be transferable to the clinical environment. J Perinatol: official journal of the California Perinatal Association.

[34] Finan E, Bismilla Z, Whyte HE, Leblanc V, McNamara PJ (2012). High-fidelity simulator technology may not be superior to traditional low-fidelity equipment for neonatal resuscitation training. J Perinatol: official journal of the California Perinatal Association.

[35] Legoux C, Gerein R, Boutis K, Barrowman N, Plint A (2021) Retention of critical procedural skills after simulation training: a systematic review. AEM Educ Train 5(3):e10536. 10.1002/aet2.10536 10.1002/aet2.10536 PMC8166305 34099989

[36] Howard S, Gaba D, Fish K, Yang G, Sarnquist F (1992). Anesthesia crisis resource management training: teaching anesthesiologists to handle critical incidents. Aviat Space Environ Med.

[37] Yang C, Yen Z, McGowan J, Chen H, Chiang W, Mancini M, Soar J, Lai M, Ma M (2012). A systematic review of retention of adult advanced life support knowledge and skills in healthcare providers. Resuscitation.

[38] Ansquer R, Mesnier T, Farampour F, Oriot D, Ghazali D (2019). Long-term retention assessment after simulation-based-training of pediatric procedural skills among adult emergency physicians: a multicenter observational study. BMC Med Educ.

[39] Bridge P, Adeoye J, Edge CN, Garner VL, Humphreys A-L, Ketterer S-J, Linforth JG, Manning-Stanley AS, Newsham D, Prescott D, Pullan SJ, Sharp J (2022). Simulated placements as partial replacement of clinical training time: a Delphi consensus study. Clin Simul Nurs.

[40] Iyer M, Way D, Schumacher D, Lo C, Leslie L (2021). How general pediatricians learn procedures: implications for training and practice. Med Educ Online.

[41] KIDS NTS team KIDS NTS. Available at: https://kids.bwc.nhs.uk/. Accessed 18 Apr 2023

[42] Bayerl R (2007) Das Muenchner Kindernotarztsystem. 1553 Einsätze aus zwei der vier Kinderkliniken in den Jahren 1998–2000. Dissertation, Medizinischen Fakultät der Ludwig-Maximilians-Universität zu München

[43] Stenke C (2004) Der Münchner Kindernotarzt. 3667 Kindernotarzteinsätze der Jahre 1998 - 2001 untersucht an zwei der vier beteiligten Kliniken. Dissertation, Universität München

